# SIRT in 2025

**DOI:** 10.1007/s00270-022-03228-6

**Published:** 2022-08-08

**Authors:** Francesca Romana Ponziani, Francesco Santopaolo, Alessandro Posa, Maurizio Pompili, Alessandro Tanzilli, Marta Maestri, Maria Pallozzi, Francesca Ibba, Riccardo Manfredi, Antonio Gasbarrini, Roberto Iezzi

**Affiliations:** 1 Dipartimento di Scienze Mediche e Chirurgiche, U.O.C. Medicina Interna e Gastroenterologia, Fondazione Policlinico Universitario, A. Gemelli IRCCS, L.go A. Gemelli 8, 00168 Rome, Italy; 2 Dipartimento di Diagnostica per Immagini, Radioterapia Oncologica ed Ematologia–U.O.C. Radiologia Diagnostica e Interventistica Generale, Fondazione Policlinico Universitario A. Gemelli IRCCS, L.go A. F.Vito 1 Gemelli 8, 00168 Rome, Italy; 3 Università Cattolica del Sacro Cuore, L.go A. Gemelli 8, 00168 Rome, Italy

**Keywords:** SIRT, Locoregional treatment, HCC, TARE, Yttrium-90, TKI, Chemotherapy, Immunotherapy

## Abstract

Selective internal radiation therapy represents an endovascular treatment option for patients with primary liver malignancies, in different clinical stages. Potential applications of this treatment are in early-stage hepatocellular carcinoma, as a curative option, or in combination with systemic treatments in intermediate and advanced-stages. This review, based on existing literature and ongoing trials, will focus on the future of this treatment in patients with hepatocellular carcinoma, in combination with systemic treatments, or with the use of new devices and technological developments; it will also describe new potential future indications and structural and organizational perspectives.

## Introduction

Selective internal radiation therapy (SIRT) is a locoregional treatment for primary and secondary liver neoplasms which applies high radiation energy selectively targeting tumor tissue, sparing the surrounding parenchyma. SIRT is mostly performed using glass or resin microparticles loaded with Yttrium-90 (90Y), and its role is well known in intermediate and advanced HCC, particularly in patients with portal vein thrombosis [1]; however, according to the recent update of Barcelona Clinic Liver Cancer Criteria, SIRT may be administrated also in BCLC stage 0 patients as an alternative to percutaneous ablation, with a curative intent, especially in elderly patients with contraindications for surgery or in patients with nodules difficult to treat with other techniques [2]; moreover in BCLC stage A patients in case of a solitary tumor or as a second choice if ablation or resection could not be performed or as a bridge treatment before surgery [3]. In the last few years SIRT has demonstrated a safety and efficacy profile comparable with transarterial chemoembolization or even superior in terms of time to progression of the disease in advanced tumors [2, 4]. In addition, radiation lobectomy can be considered to induce liver tissue hypertrophy before surgery, and also to control tumor progression as a bridge to liver transplantation [4]. In advanced HCC, SIRT will be combined with systemic treatment such as tyrosine kinase inhibitors, immunotherapies, or both [2].

This review will focus on available data and ongoing trials on the future applications as well as structural and organizational perspectives of SIRT, exploring new potential combined treatment options as well as new devices, technological developments, that will allow potential new indications.

## Combination of TARE with TKI Medication

Radiation therapy (RT) is efficient in an oxygenated environment, as the production of reactive oxygen species causes cell death; conversely, hypoxic conditions determine radiation-resistance. RT (particularly fractionated RT) enhances the production of Hypoxia Inducible Factor (HIF), Vascular Endothelial Growth Factor (VEGF), Platelet-derived Growth Factor (PDGF), Fibroblast Growth Factor (FGF), and other proinflammatory cytokines, inducing vessels proliferation. Tumor cells escape from hypoxia producing VEGF and other cytokines, activating neoangiogenesis; however, these vessels are aberrant, leading to the maintenance of the hypoxic environment. Antiangiogenic drugs restore the radiosensitivity of tumors by remodeling vessels and causing a transient vascular normalization that provides oxygen delivery to tumor cells, whereas in the long-term the reduction of blood vessels leads to hypo-oxygenation. High radiation doses damage tumor vessels and induce endothelial cells apoptosis; antiangiogenic drugs counteracting VEGF corroborate RT efficacy [33–36]. As HCC is highly vascularized, antiangiogenic drugs such as the tyrosine kinase inhibitors (TKIs) Sorafenib and Lenvatinib are used for the first-line treatment of advanced, unresectable HCC. Regorafenib and Cabozantinib are TKIs used as second line options after progression to Sorafenib, similarly to the anti-VEGF receptor-2 monoclonal antibody Ramucirumab [37–41]. SIRT has been compared with Sorafenib in patients with advanced HCC or with locally advanced HCC after transarterial chemoembolization (TACE) failure [2, 37, 42]. The SARAH study was the first multicenter prospective phase-III trial comparing the efficacy of SIRT with Sorafenib. Patients treated with SIRT showed a better safety profile and quality of life, and higher tumor response rates (19% versus 12%, *p* = 0.0421), even in patients with portal vein invasion [43]. Analyzing the tumor recurrence rate, the SIRT group showed fewer events than the Sorafenib group and better tolerability profile, suggesting the choice of SIRT in patients with intrahepatic disease, tumor burden ≤ 25% and compensated liver function [44]. Despite these relevant results, the study did not meet the primary endpoint criteria, as OS was not different between the SIRT and Sorafenib groups. An ancillary study demonstrated a significant difference in OS in patients who received a TD ≥ 100 Gy (14.1 months) than those who received < 100 Gy (6.7 months) (*p* = 0.001), with 74% of disease control in the first ones; no differences in adverse events were described [45]. A prospective multicenter trial in the Asian population obtained the same results of the SARAH trial, demonstrating better local tumor control, safety and tolerability profile versus systemic therapy and tumor response rate of 16.5% in the RE group versus 1.7% in the TKI group (*p* < 0.001) in patients with BCLC B/C HCC, but without significant benefits on OS and progression-free survival (PFS) [46].

Considering the SIRT local tumor control and the modulation on inflammation and neoangiogenesis of anti-VEGF therapies that may overcome radiation resistance, several studies evaluated the combination of SIRT and TKIs in patients with advanced HCC [33–36].

The SORAMIC study was prospectively designed to evaluate if the combination of SIRT plus Sorafenib would improve OS versus Sorafenib monotherapy in patients with advanced HCC [47]. The results were similar to previous studies: OS was 14 months for SIRT versus 11 months for Sorafenib; the subgroups evaluation showed better OS in patients ≤ 65 years, in non-cirrhotic or compensated non-alcoholic cirrhotic patients, and in patients with more than seven nodules. Previous TACE was associated with better survival in the Sorafenib arm [47, 48]. The SORAMIC trial also evaluated the alteration of liver enhancement after gadoxetic acid administration during hepatobiliary phase of magnetic resonance imaging (MRI) compared to the spleen enhancement; the Liver-to-Spleen ratio (LSR) directly correlates with reduced liver function: a low LSR was described in the presence of higher levels of AST, bilirubin, ascites and varices [49]. Extrahepatic disease spread did not significantly impact on survival between the two groups (*p* = 0.6483), nor the progression during the study (19% of cases); conversely, lung metastases reduced patients’ survival in both groups (*p* = 0.0060). Therefore, except for lung metastases, presence of extrahepatic metastases in patients with a high HCC liver burden should not affect the possibility to perform locoregional treatments to control the intrahepatic disease, as the main cause of death in these patients is intrahepatic progression and liver failure [50, 51]. A meta-analysis of three studies revealed the non-inferiority of SIRT compared to Sorafenib for the treatment of advanced HCC [43, 46, 47]. SIRT led to a better OS in patients with chronic hepatitis B or in non-cirrhotic HCC patients. Furthermore, a higher percentage of partial responses (PR) was observed in the SIRT arm, while patients in the Sorafenib group frequently showed disease stability (DS) [52]. However, these studies did not address the delivered TD, which could have affected the final results [53, 54].

A phase-II study determined safety and efficacy of Sorafenib followed by SIRT in patients with advanced or metastatic HCC and Child–Pugh A, naive to locoregional treatments or who were unsuccessfully treated: 35.7% of patients presented PR, 47% DS, whereas none achieved complete response (CR). Median PFS was 10.3 months; OS was 13.2 months [55].

## Combination of TARE with Immunotherapy

Immune checkpoint inhibitors (ICIs) represent the new frontier in cancer therapy [56–61]. They are antibodies targeting proteins called “immune checkpoints”, such as programmed death-1 (PD-1), programmed death-ligand 1 (PD-L1) and cytotoxic T-lymphocyte associated protein-4 (CTLA-4), which are present on T-cells, B-cells and antigen presenting cells (APCs), involved in maintenance of self-tolerance. Cancer cells implement evasion mechanisms of immune checkpoints hyperexpression to escape immune response. Blocking immune checkpoints enhances immune response and promotes anticancer defense. ICIs are used as first- or second-line therapy, often in combination with TKIs or other antiangiogenic drugs [62].

RT boosts inflammation, leading to a systemic response that may result in anti-tumor effects in sites distant from the irradiated area, the so-called “abscopal effect”, linked to “immune cell death” [63]. Stress derived by RT enhances damage-associated molecular patterns (DAMPs) expression and recruiting of immune cells. RT damage on endoplasmic reticulum and cellular or nuclear membranes, causes the activation of calreticulin and its exposition on the cell membrane, leading to dendritic cells (DCs) activation, phagocytosis of tumor cells and antigen presentation to cytotoxic lymphocytes [64, 65]. Dying tumor cells also release antigens, as DNA, that stimulate immunological response [64].

Therefore, combination of RT and ICIs is expected to obtain promising results. ICIs need appropriate antigen presentation to be effective, a process enhanced by RT. On the other hand, RT also causes upregulation of immune checkpoints, generating radiation-resistance; thus, ICIs can restore the cytotoxic T-cell and APCs activities, and may implement the abscopal effect, overcoming the radiation-resistance [66–68]. SIRT determines a significant shift in the characteristics of tumor infiltrating lymphocytes (TIL), increasing CD56 + natural killer (NK), CD4 + and CD8 + T cells [69]. Conversely, before SIRT, TIL were mostly regulatory T-cells that downregulated the immune response against tumors. Tumor necrosis factor (TNF)-alpha was elevated in SIRT responders, and a sustained response was found when TIL expressed higher levels of PD-1 before and after SIRT [70].

Indeed, as described by previous studies, following radioembolization it is reported an increased production of inflammatory cytokines, such as interleukin (IL) 1, IL-6 and IL-8, TNFα, and the release of several inducible factor as hypoxia inducible factor 1α, VEGF, matrix metalloproteinases (MMPs), and mammalian target of rapamycin (mTOR). The combination of SIRT with immune checkpoint inhibitors has demonstrated to enhance the systemic inflammatory response by reverting the suppressive phenotype derived by the upregulation of tumor induced immune checkpoints on peripheral and intratumoral immune cells and stimulating them to produce TNF α and granzyme B that lead to a sustained systemic inflammation and an increased anti-tumor response [13, 71, 72].

Given their synergistic immunomodulatory effects, association of SIRT and immunotherapy has been tested in several clinical trials [69].

In a retrospective study, patients with advanced or intermediate-stage HCC and good liver function (Child–Pugh A-B7) were treated with Nivolumab or Nivolumab plus Ipilimumab after SIRT: two patients experienced delayed grade 3/4 hepatobiliary toxicity [71]. Another retrospective study evaluated patients with BCLC B/C HCC, prevalently Child–Pugh A, who underwent SIRT or TACE after Nivolumab: two patients discontinued treatment due to immune-related adverse events (irAEs) (pneumonitis and transaminitis), five grade 3/4 hepatobiliary toxicity occurred within 3 months after locoregional therapy, with no grade 3/4 adverse events attributable to Nivolumab. 1-month overall objective response rate (ORR) was 45% [72].

A prospective phase-I clinical trial evaluated the combination of Nivolumab plus SIRT in patients with advanced HCC not eligible for surgical treatments. Nivolumab was started after SIRT at the dose of 80 mg (group 1) or 240 mg (group 2); primary endpoint was defining the maximum tolerated dose of Nivolumab when combined with SIRT. Group 2 dose was well-tolerated; the most relevant irAE in both groups was grade 1–2 transaminases elevation. Overall disease control (ODC) rate was 82%; 9 out of 11 patients showed stable disease [73]. A phase-II clinical trial enrolled patients with advanced HCC and Child–Pugh A cirrhosis not eligible for surgery, who underwent SIRT followed by Nivolumab administration, obtaining 30.6% ORR (1 CR, 10 PR); five patients presented serious treatment-related adverse events (Steven–Johnson syndrome, hepatitis E infection, fever, liver abscesses, ascites) [74]*.*

Several ongoing studies are evaluating the safety and efficacy of SIRT plus ICIs. A phase-I study [NCT03812562] is evaluating the combination of Nivolumab plus SIRT after surgical resection; primary endpoint is recurrence rate. NCT03099564 is evaluating the combination of Pembrolizumab plus SIRT in patients with HCC not eligible for surgical resection or liver transplantation; primary endpoint is 6-months PFS.

Combination of Durvalumab, an anti PD-L1 antibody, and Tremelimumab, an anti CTLA-4 antibody, was superior to Sorafenib in terms of OS either in combination or as Durvalumab monotherapy [61, 75]. A phase-Ib trial [/NCT04605731] will evaluate the safety of Durvalumab plus Tremelimumab or Durvalumab alone after SIRT in patients with unresectable locally advanced BCLC B/C HCC with Child–Pugh A and tumor burden < 50% in terms of ORR according to RECIST, mRECIST and immune mRECIST criteria. A multicenter randomized phase-II trial [NCT05063565] will evaluate the efficacy of SIRT plus combination of Durvalumab and Tremelimumab versus SIRT alone in terms of ORR and response duration in naive HCC patients not eligible for (or who refused) curative treatments. A phase-II randomized trial [NCT04522544] will investigate safety and efficacy of Durvalumab plus Tremelimumab after TACE or SIRT in patients with multifocal HCC or with a single nodule not eligible for curative treatments, or with hepatic veins or portal vein involvement. Another phase-I/II trial [NCT0412499] will investigate if combination of Durvalumab plus SIRT can improve time to progression (TTP) in locally advanced unresectable HCC. A multicenter randomized phase-II trial [NCT04541173] is evaluating patients with Child–Pugh A and BCLC B HCC not eligible for surgical treatments that will receive SIRT alone or followed by Atezolizumab plus Bevacizumab; primary endpoint is 1-year PFS.

## Potential New Isotopes

Currently, the most used radiopharmaceutical product for transarterial radioembolization (TARE) consists of 90Y microspheres available in two formulations: the glass-based TheraSphere (BTG, Ontario, Canada) and resin-based SIR-Spheres® (SIRTex, North Sydney, Australia) microspheres [76]. Unfortunately, there are some limits concerning 90Y utilization: microspheres production is a high-cost multi-step process, since 90Y derives from strontium-90 (90Sr), which is a fission product of uranium in nuclear reactors; this process needs high specialized personnel and brings a heavy radioactive environmental burden [77]. In addition, 90Y is a pure therapeutic beta-energy emitter, which makes the evaluation of radiation dosimetry and post-TARE microspheres distribution in tissues difficult to be detected, because of intrinsic properties of beta rays, not suitable for diagnostic imaging. For these reasons, in the last few decades new microspheres labeled with 166Ho have been developed [26]. 166Ho TARE seems to be a feasible option for HCC treatment, with a good safety and toxicity profile, as well as for patients with unresectable and chemo-resistant liver metastases [78, 79]. Compared with 90Y, 166Ho has the advantage of possessing a *γ* emission (81 keV) suitable for SPECT imaging. Moreover, holmium is highly paramagnetic, thus enabling MRI imaging and quantification. Van Roekel et al. found that patient survival was significantly longer in case of a mean-tumor absorbed dose greater than 90 Gy in case of 166Ho TARE [80].

Another interesting isotope which is gaining interest among interventional radiologists is Samarium-153 (153Sm) [81]. 153Sm is a radionuclide derived from purification and neutron activation of 152Sm. It has a half-life of 46.3 h and emits beta rays of 0.81 MeV (20%), 0.71 MeV (30%), and 0.64 MeV (50%), with maximum penetration in soft tissue up to 4.0 mm; moreover, 153Sm releases gamma particles of 103 keV that may be utilized for scintigraphy imaging and single-photon emission computed tomography (SPECT) and it has a thermal neutron activation cross section of 210 barns [82]. Neutron activation has a lower cost of production compared to nuclear fission and may be more available worldwide. During neutron activation, the 152Sm atoms absorb one neutron from the thermal neutron flux to become 153Sm, with consequent release of energy in the form of gamma radiation. Neutron irradiation may last at maximum of 6 h; longer processes cause radionuclide impurities production. In one study, the radioactive microspheres with size of 20–40 μm were produced and bound to Amberlite cation exchange resin but they resulted inappropriate and irregular for shape and presented a high rate of fragmentation during the neutron activation process [83]. In another study, 152Sm chloride hexahydrate and 152-Sm carbonate have been used to obtain 35 μm diameter resin microspheres; they resulted able to preserve their shape and integrity during the neutron activation process showing a better efficiency (97–99%) than 153Sm-labeled microspheres (85–97%) [84]. The same research group in recent years formulated a new type of poly-l-lactic acid microspheres (PLLA) incorporated with 152Sm acetylacetonate [85]. In another study, 153Sm oxide-loaded polystyrene microspheres were developed, and they had a remarkable retention efficiency in both saline solution and blood plasma with a medium duration of 550 h [86]. Since no ionizing radiation is needed for the production, these microspheres may be synthetized in a standard chemistry laboratory and then they may be sent in a specialized center to be activated and obtain radioactive 153Sm oxide-loaded polystyrene microspheres. Previously to 153Sm, others neutron activated radionuclides such as Holmium and Rhenium were tested as possible alternatives to 90Y, but they were excluded because of their short half-lives and the need of elevated neutron flux reactors compared to 153Sm [86, 87]. 153Sm is a promising “theranostic” (therapeutic and diagnostic) agent, suitable for a combinatory diagnostic and therapeutical approach, but further studies are needed to better delineate its cytotoxicity and its efficiency in comparison to 90Y microspheres.

## Potential New Indications: Outside the Liver

Interest in applications of TARE outside of the liver is emerging and small initial studies have been performed primarily in animal models to assess the effects of TARE on other organs, such as brain.

The standard of care for Glioblastoma multiforme (GBM), a malignant brain tumor, is surgical resection followed by adjuvant chemotherapy [88]. GBM local recurrence, even with treatment, is common due to tumoral cell infiltration [89]. Radiation therapy is an important tool for newly diagnosed GBM and is commonly performed using external beam radiation therapy (EBRT), which provides little neurotoxicity [90, 91]. Other options are brachytherapy and stereotactic radiosurgery. TARE, which is commonly used for the treatment of liver cancer, delivers much more radiation dose in hypervascular tumors compared to EBRT, and reduces nontarget radiation dose [92]. Using TARE for intra-axial brain tumors could be problematic due to the potential ischemic changes induced by microspheres in the normal brain tissue. The potential effectiveness of TARE for the treatment of GBM could be based on the balance of ischemic effects, delivered radiation dose within the tumor, and delivered radiation dose to healthy brain tissue. A recent paper evaluated the safety, feasibility, and efficacy of 90Y TARE for the treatment of spontaneous brain cancers in a canine model [93]. In this study, three healthy research dogs and five patient dogs affected by spontaneous intra-axial brain masses underwent cerebral 90Y TARE using glass microspheres (TheraSphere). Post-treatment PET-CT and neurological examinations by veterinary neurologists were performed. Research dogs were euthanized after 1 month and the brains were extracted and analyzed (micro-dosimetry and histopathologic analyses); on the other hand, patient dogs underwent post-treatment MRI at 1-, 3-, and 6-months with a long-term follow-up. 1 month after treatment, research dog pathologic analysis revealed no evidence of atrophy and rare foci of chronic infarcts. Absorbed doses to masses in patient dogs ranged from 45.4, to 64.1 Gy and the dose to healthy brain tissue was from 15.4 to 33.3 Gy. Among both groups (patient and research dogs), six developed acute transient neurologic deficits after the treatment. At 1 month follow-up, patient dogs showed a 24–94% reduction in tumor volume, achieving a partial response in 3 of them at 6 months follow-up. This preliminary study in dogs underlines the feasibility and safety of 90Y TARE as a potential treatment for brain cancer.

In 2001 van Es et al. published a study in which 22 rabbits with VX2 squamous cell carcinomas implanted into the auricles were treated with TARE using radioactive or inactive holmium-labeled poly-(L-lactic acid) (HoPLA) microspheres, achieving a complete response in 79% and 86% following embolization with radioactive and inactive microspheres, respectively [94]. More than 95% of the microspheres were retained within the tumor. TARE with 166HoPLA microspheres could be a promising treatment for unresectable head-and-neck cancer but further studies on humans are needed.

Another potential new indication would be related to prostate diseases, in case of malignancy or also benign hyperplasia. In detail, as reported in the paper of Mouli et al. performed in a canine model, prostate 90Y TARE seems to be safe and feasible, leading to focal dose-dependent changes in the gland, such as atrophy and focal necrosis, without inducing unwanted extra-prostatic effects [95].

## Structural and Organizational Perspectives

Office-based interventional oncology (IO) offers great benefits compared with hospital-based IO, as a more comfortable environment and greater convenience for patients. The outpatient setting allows for patient-focused services, faster check-in, less paperwork, and efficient postprocedural management/discharge, with greater patient satisfaction [96]. Physician benefits include better work-life balance, more manageable hours, no call or weekend obligations.

The increasing burden of IO procedures, pressure to reduce costs, and patients’ wishes – particularly due to pandemic conditions – has stimulated the development of ambulatory care for many procedures historically performed in the hospital, such as liver-directed therapies.

SIRT is characterized by potential adverse events ranging from acute (post-embolization syndrome, pain, vomiting, nausea, fever, leucocytosis, cholecystitis, pancreatitis) to delayed ones (gastro-duodenal inflammation, ulceration, bleeding, pneumonia); however, the most frequent ones are represented by constitutional symptoms, usually lasting for 1 week, not requiring hospitalization, treated with medications [97–106].

RE procedures can be safely and effectively performed on an outpatient basis; Aberle et al. retrospectively evaluated 212 patients treated with SIRT for primary and secondary malignancies, with only a 3.3% of adverse events requiring hospitalization and a very low radiation exposure [107, 108]. These advantages could be improved with the use of transradial approach, characterized by a less postprocedural discomfort at the access site, and reduced limitations in patient’s basic activities, leading to faster discharge [109, 110].

Careful selection of patients is mandatory, based on medical (comorbidities and risk factors) and sociological (compliance, social and family situation, access to medical care, available home aid) criteria.

The possibility to perform a single-day SIRT reduced the disadvantages of RE (typically requiring at least two visits) compared to other locoregional therapies: RE can be safely performed with pre-treatment diagnostic angiography, dosimetry evaluation, and therapeutic SIRT in the same day [111–116].

Dosimetry measurements can deliver personalized and optimized dose to the tumor in TARE treatments, with both increase in treatment safety and efficacy. 99mTc-MAA SPECT represents the current standard for "scout" dosimetry; however, research in this field is constantly evolving and refining, with alternative particles tested in clinical studies: in particular, the use of the same particle in both scout and treatment procedure could grant better accuracy in dose delivery than MAA [117, 118]. Safety and effectiveness of 166Ho use as a “scout” dose, have been evaluated by various studies, concluding that 166Ho can be used as an alternative to 99mTc-MAA, with a greater predictive value in evaluating lung shunt presence, more reliable pre-treatment imaging and better agreement between scout and treatment volumes [119, 120].

Bakker et al. demonstrated how Holmium-166 (166Ho) microspheres used for RE can be accurately detected at postprocedural CT scan, being an alternative to SPECT evaluation, leading to faster patient discharge [121, 122].

Performing an outpatient single-day procedure, SIRT could become even more competitive with other locoregional therapies, beneficial for patients with travel hardships, difficult vascular access, contrast medium allergies, resulting in cost savings and fewer complications, becoming an attractive care model and an opportunity to mitigate infection risk and logistical challenges associated with COVID-19 pandemic [115].

## Conclusions

Radioembolization is a minimally invasive procedure with an established role in the management of primary and secondary hepatic tumors, providing personalized treatment approaches with palliative and curative indications. Recent advancements and new techniques led to its application across the Barcelona Clinic Liver Cancer staging paradigm, as a curative treatment or as a bridge or downstage to liver transplantation. Great improvement in liver cancer treatment will also be granted by combined application of radioembolization and systemic or immunotherapy, with the possibility to be performed in an outpatient single-day setting. Appropriate patient selection, comprehensive work-up and multidisciplinary tumor board evaluation remain the main preprocedural criteria to offer an effective and safe treatment, improving clinical outcome and patient survival. Innovative devices, new techniques as well as technological developments will also allow to expand its clinical indications beyond the liver.

## References

[1] Mazzaferro V, Sposito C, Bhoori S, Romito R, Chiesa C, Morosi C, Maccauro M, Marchianò A, Bongini M, Lanocita R, Civelli E, Bombardieri E, Camerini T, Spreafico C (2013). Yttrium-90 radioembolization for intermediate-advanced hepatocellular carcinoma: a phase 2 study. Hepatology.

[2] Reig M, Forner A, Rimola J, Ferrer-Fàbrega J, Burrel M, Garcia-Criado Á, Kelley RK, Galle PR, Mazzaferro V, Salem R, Sangro B, Singal AG, Vogel A, Fuster J, Ayuso C, Bruix J (2021). BCLC strategy for prognosis prediction and treatment recommendation: the 2022 update. J Hepatol..

[3] Biederman DM, Titano JJ, Bishay VL, Durrani RJ, Dayan E, Tabori N, Patel RS, Nowakowski FS, Fischman AM, Kim E (2017). Radiation segmentectomy versus TACE combined with microwave ablation for unresectable solitary hepatocellular carcinoma Up to 3 cm: a propensity score matching study. Radiology.

[4] Van Cutsem E, Cervantes A, Adam R, Sobrero A, Van Krieken JH, Aderka D, Aranda Aguilar E, Bardelli A, Benson A, Bodoky G, Ciardiello F, D'Hoore A, Diaz-Rubio E, Douillard JY, Ducreux M, Falcone A, Grothey A, Gruenberger T, Haustermans K, Heinemann V, Hoff P, Köhne CH, Labianca R, Laurent-Puig P, Ma B, Maughan T, Muro K, Normanno N, Österlund P, Oyen WJ, Papamichael D, Pentheroudakis G, Pfeiffer P, Price TJ, Punt C, Ricke J, Roth A, Salazar R, Scheithauer W, Schmoll HJ, Tabernero J, Taïeb J, Tejpar S, Wasan H, Yoshino T, Zaanan A, Arnold D (2016). ESMO consensus guidelines for the management of patients with metastatic colorectal cancer. Ann Oncol..

[5] Vitale A, Burra P, Frigo AC, Trevisani F, Farinati F, Spolverato G, Volk M, Giannini EG, Ciccarese F, Piscaglia F, Rapaccini GL (2015). Survival benefit of liver resection for patients with hepatocellular carcinoma across different Barcelona Clinic Liver Cancer stages: a multicentre study. J hepatol..

[6] Heil J, Schadde E (2021). Simultaneous portal and hepatic vein embolization before major liver resection. Langenbecks Arch Surg..

[7] de Graaf W, van den Esschert JW, van Lienden KP, van Gulik TM (2009). Induction of tumor growth after preoperative portal vein embolization: is it a real problem?. Ann Surg Oncol..

[8] Hayashi S, Baba Y, Ueno K, Nakajo M, Kubo F, Ueno S, Aikou T, Komokata T, Nakamura N, Sakata R (2007). Acceleration of primary liver tumor growth rate in embolized hepatic lobe after portal vein embolization. Acta Radiol..

[9] Vouche M, Lewandowski RJ, Atassi R, Memon K, Gates VL, Ryu RK, Gaba RC, Mulcahy MF, Baker T, Sato K, Hickey R, Ganger D, Riaz A, Fryer J, Caicedo JC, Abecassis M, Kulik L, Salem R (2013). Radiation lobectomy: time-dependent analysis of future liver remnant volume in unresectable liver cancer as a bridge to resection. J Hepatol..

[10] Edeline J, Lenoir L, Boudjema K, Rolland Y, Boulic A, Le Du F, Pracht M, Raoul JL, Clément B, Garin E, Boucher E (2013). Volumetric changes after (90)y radioembolization for hepatocellular carcinoma in cirrhosis: an option to portal vein embolization in a preoperative setting?. Ann Surg Oncol..

[11] Cucchetti A, Cappelli A, Ercolani G, Mosconi C, Cescon M, Golfieri R, Pinna AD (2016). Selective internal radiation therapy (SIRT) as conversion therapy for unresectable primary liver malignancies. Liver Cancer..

[12] Fernández-Ros N, Silva N, Bilbao JI, Iñarrairaegui M, Benito A, D'Avola D, Rodriguez M, Rotellar F, Pardo F, Sangro B (2014). Partial liver volume radioembolization induces hypertrophy in the spared hemiliver and no major signs of portal hypertension. HPB (Oxford).

[13] Taylor AC, Maddirela D, White SB (2019). Role of radioembolization for biliary tract and primary liver cancer. Surg Oncol Clin N Am..

[14] Theysohn JM, Ertle J, Müller S, Schlaak JF, Nensa F, Sipilae S, Bockisch A, Lauenstein TC (2014). Hepatic volume changes after lobar selective internal radiation therapy (SIRT) of hepatocellular carcinoma. Clin Radiol..

[15] Birgin E, Rasbach E, Seyfried S, Rathmann N, Diehl SJ, Schoenberg SO, Reissfelder C, Rahbari NN (2020). Contralateral liver hypertrophy and oncological outcome following radioembolization with 90Y-microspheres: a systematic review. Cancers (Basel)..

[16] Salem R, Johnson GE, Kim E, Riaz A, Bishay V, Boucher E, Fowers K, Lewandowski R, Padia SA (2021). Yttrium-90 radioembolization for the treatment of solitary. Unresectable HCC: LEGACY Study Hepatology..

[17] Gaba RC, Lewandowski RJ, Kulik LM, Riaz A, Ibrahim SM, Mulcahy MF, Ryu RK, Sato KT, Gates V, Abecassis MM, Omary RA, Baker TB, Salem R (2009). Radiation lobectomy: preliminary findings of hepatic volumetric response to lobar yttrium-90 radioembolization. Ann Surg Oncol..

[18] Liebl M, Pedersoli F, Zimmermann M, Schulze-Hagen M, Truhn D, Sieben P, von Stillfried S, Tschinaev A, Heinzel A, Kuhl CK, Bruners P, Isfort P (2021). Induction of contralateral hepatic hypertrophy by unilobar yttrium-90 transarterial radioembolization versus portal vein embolization: an animal study. J Vasc Interv Radiol..

[19] Kutlu R, Karatoprak S (2020). Radioembolization for hepatocellular carcinoma in downstaging and bridging for liver transplantation. J Gastrointest Cancer..

[20] Samim M, Lam MGEH (2017). Safety and efficacy of Y-90 radioembolization after prior major hepatic resection: dosimetric consideration. Cardiovasc Intervent Radiol..

[21] Semaan S, Makkar J, Lewis S, Chatterji M, Kim E, Taouli B (2017). Imaging of hepatocellular carcinoma response after 90Y RADIOEMBOLIZATION. AJR Am J Roentgenol..

[22] Palard X, Edeline J, Rolland Y, Le Sourd S, Pracht M, Laffont S, Lenoir L, Boudjema K, Ugen T, Brun V, Mesbah H, Haumont LA, Loyer P, Garin E (2018). Dosimetric parameters predicting contralateral liver hypertrophy after unilobar radioembolization of hepatocellular carcinoma. Eur J Nucl Med Mol Imaging..

[23] Fernandez-Ros N, Iñarrairaegui M, Paramo JA, Berasain C, Avila MA, Chopitea A, Varo N, Sarobe P, Bilbao JI, Dominguez I, D'Avola D, Herrero JI, Quiroga J, Sangro B (2015). Radioembolization of hepatocellular carcinoma activates liver regeneration, induces inflammation and endothelial stress and activates coagulation. Liver Int..

[24] Gabr A, Riaz A, Johnson GE, Kim E, Padia S, Lewandowski RJ, Salem R (2021). Correlation of Y90-absorbed radiation dose to pathological necrosis in hepatocellular carcinoma: confirmatory multicenter analysis in 45 explants. Eur J Nucl Med Mol Imaging..

[25] Qadan M, Fong ZV, Delman AM, Gabr A, Salem R, Shah SA (2021). Review of use of Y90 as a bridge to liver resection and transplantation in hepatocellular carcinoma. J Gastrointest Surg..

[26] Reinders MTM, Smits MLJ, van Roekel C, Braat AJAT (2019). Holmium-166 microsphere radioembolization of hepatic malignancies. Semin Nucl Med..

[27] Depalo T, Boni G, Ghinolfi D, Bozzi E, Cervelli R, Catalano G, Volterrani D, Bargellini I (2021). Potential benefits of holmium-166 radioembolization as a neoadjuvant treatment of intrahepatic cholangiocarcinoma. Cardiovasc Intervent Radiol..

[28] Riby D, Mazzotta AD, Bergeat D, Verdure L, Sulpice L, Bourien H, Lièvre A, Rolland Y, Garin E, Boudjema K, Edeline J (2020). Downstaging with radioembolization or chemotherapy for initially unresectable intrahepatic cholangiocarcinoma. Ann Surg Oncol..

[29] Edeline J, Touchefeu Y, Guiu B, Farge O, Tougeron D, Baumgaertner I, Ayav A, Campillo-Gimenez B, Beuzit L, Pracht M, Lièvre A, Le Sourd S, Boudjema K, Rolland Y, Boucher E, Garin E (2020). Radioembolization plus chemotherapy for first-line treatment of locally advanced intrahepatic cholangiocarcinoma: a phase 2 clinical trial. JAMA Oncol..

[30] Tabone M, Calvo A, Russolillo N, Langella S, Carbonatto P, Lo Tesoriere R, Richetta E, Pellerito R, Ferrero A (2020). Downstaging unresectable hepatocellular carcinoma by radioembolization using 90-yttrium resin microspheres: a single center experience. J Gastrointest Oncol..

[31] Tohme S, Sukato D, Chen HW, Amesur N, Zajko AB, Humar A, Geller DA, Marsh JW, Tsung A (2013). Yttrium-90 radioembolization as a bridge to liver transplantation: a single-institution experience. J Vasc Interv Radiol..

[32] Bilbao JL, Iezzi R, Goldberg SN, Sami A, Akhan O, Giuliante F, Pompili M, Crocetti L, Malagari K, Valentini V, Gasbarrini A, Colosimo C, Manfredi R (2017). The ten commandments of hepatic radioembolization: expert discussion and report from mediterranean interventional oncology (MIOLive) congress 2017. Eur Rev Med Pharmacol Sci..

[33] Shannon AM, Williams KJ (2008). Antiangiogenics and radiotherapy. J Pharm Pharmacol..

[34] Marques FG, Poli E, Malaquias J, Carvalho T, Portêlo A, Ramires A, Aldeia F, Ribeiro RM, Vitorino E, Diegues I, Costa L (2019). Low doses of ionizing radiation activate endothelial cells and induce angiogenesis in peritumoral tissues. Radiother Oncol..

[35] Kanthou C, Tozer G (2019). Targeting the vasculature of tumours: combining VEGF pathway inhibitors with radiotherapy. Br J Radiol..

[36] Goedegebuure RSA, de Klerk LK, Bass AJ, Derks S, Thijssen VLJL (2019). Combining radiotherapy with anti-angiogenic therapy and immunotherapy; A therapeutic triad for cancer?. Front Immunol..

[37] Llovet JM, Ricci S, Mazzaferro V, Hilgard P, Gane E, Blanc J-F, Cosme A, de Oliveira A, Santoro J-LR, Forner A, Schwartz M, Porta C, Zeuzem S, Bolondi L, Greten TF, Galle PR, Seitz J-F, Borbath I, Häussinger D, Giannaris T, Shan M, Moscovici M, Voliotis D, Bruix J (2008). Sorafenib in advanced hepatocellular carcinoma. N Engl J Med..

[38] Kudo M, Finn RS, Qin S, Han KH, Ikeda K, Piscaglia F, Baron A, Park JW, Han G, Jassem J, Blanc JF, Vogel A, Komov D, Evans TRJ, Lopez C, Dutcus C, Guo M, Saito K, Kraljevic S, Tamai T, Ren M, Cheng AL (2018). Lenvatinib versus sorafenib in first-line treatment of patients with unresectable hepatocellular carcinoma: a randomised phase 3 non-inferiority trial. Lancet.

[39] Bruix J, Qin S, Merle P, Granito A, Huang YH, Bodoky G, Pracht M, Yokosuka O, Rosmorduc O, Breder V, Gerolami R (2017). Regorafenib for patients with hepatocellular carcinoma who progressed on sorafenib treatment (RESORCE): a randomised, double-blind, placebo-controlled, phase 3 trial. Lancet.

[40] Abou-Alfa GK, Meyer T, Cheng AL, El-Khoueiry AB, Rimassa L, Ryoo BY, Cicin I, Merle P, Chen Y, Park JW, Blanc JF, Bolondi L, Klümpen HJ, Chan SL, Zagonel V, Pressiani T, Ryu MH, Venook AP, Hessel C, Borgman-Hagey AE, Schwab G, Kelley RK (2018). Cabozantinib in patients with advanced and progressing hepatocellular carcinoma. N Engl J Med..

[41] Zhu AX, Kang YK, Yen CJ, Finn RS, Galle PR, Llovet JM, Assenat E, Brandi G, Pracht M, Lim HY, Rau KM (2019). Ramucirumab after sorafenib in patients with advanced hepatocellular carcinoma and increased α-fetoprotein concentrations (REACH-2): a randomised, double-blind, placebo-controlled, phase 3 trial. Lancet Oncol..

[42] Galle PR, Forner A, Llovet JM, Mazzaferro V, Piscaglia F, Raoul Jean-L, Schirmacher P, Vilgrain V (2018). EASL clinical practice guidelines: management of hepatocellular carcinoma. J Hepatol..

[43] Vilgrain V, Pereira H, Assenat E, Guiu B, Ilonca AD, Pageaux G-P, Sibert A, Bouattour M, Lebtahi R, Allaham W, Barraud H, Laurent V, Mathias E, Bronowicki J-P, Tasu J-P, Perdrisot R, Silvain C, Gerolami R, Mundler O, Seitz J-F, Vidal V, Aubé C, Oberti F, Couturier O, Brenot-Rossi I, Raoul J-L, Sarran A, Costentin C, Itti E, Luciani A, Adam R, Lewin M, Samuel D, Ronot M, Dinut A, Castera L, Chatellier G (2017). Efficacy and safety of selective internal radiotherapy with yttrium-90 resin microspheres compared with sorafenib in locally advanced and inoperable hepatocellular carcinoma (SARAH): an open-label randomised controlled phase 3 trial. Lancet Oncol..

[44] Palmer DH, Hawkins NS, Vilgrain V, Pereira H, Chatellier G, Ross PJ (2020). Tumor burden and liver function in HCC patient selection for selective internal radiation therapy: SARAH post-hoc study. Future Oncol..

[45] Hermann AL, Dieudonné A, Ronot M, Sanchez M, Pereira H, Chatellier G, Garin E, Castera L, Lebtahi R, Vilgrain V (2020). SARAH Trial Group Relationship of tumor radiation–absorbed dose to survival and response in hepatocellular carcinoma treated with transarterial radioembolization with 90Y in the SARAH study. Radiology.

[46] Chow PKH, Gandhi M, Tan S-B, Khin MW, Khasbazar A, Janus Ong S, Choo P, Cheow PC, Chotipanich C, Lim K, Lesmana LA, Manuaba TW, Yoong BK, Raj A, Law CS, Cua IHY, Lobo RR, Teh CSC, Kim YH, Jong YW, Han H-S, Bae S-H, Yoon H-K, Lee R-C, Hung C-F, Peng C-Y, Liang P-C, Bartlett A, Kok KYY, Thng C-H, Low AS-C, Goh ASW, Tay KH, Lo RHG, Goh BKP, Ng DCE, Lekurwale G, Liew WM, Gebski V, Mak KSW, Soo KC (2018). SIRveNIB: selective internal radiation therapy versus sorafenib in asia-pacific patients with hepatocellular carcinoma. J Clin Oncol..

[47] Ricke J, Klümpen HJ, Amthauer H, Bargellini I, Bartenstein P, de Toni EN, Gasbarrini A, Pech M, Peck-Radosavljevic M, Popovič P, Rosmorduc O, Schott E, Seidensticker M, Verslype C, Sangro B, Malfertheiner P (2019). Impact of combined selective internal radiation therapy and sorafenib on survival in advanced hepatocellular carcinoma. J Hepatol..

[48] Ricke J, Schinner R, Seidensticker M, Gasbarrini A, van Delden OM, Amthauer H, Peynircioglu B, Bargellini I, Iezzi R, De Toni EN, Malfertheiner P, Pech M, Sangro B (2021). Liver function after combined selective internal radiation therapy or sorafenib monotherapy in advanced hepatocellular carcinoma. J Hepatol..

[49] Öcal O, Peynircioglu B, Loewe C, van Delden O, Vandecaveye V, Gebauer B, Zech CJ, Sengel C, Bargellini I, Iezzi R, Benito A, Schütte K, Gasbarrini A, Seidensticker R, Wildgruber M, Pech M, Malfertheiner P, Ricke J, Seidensticker M (2022). Correlation of liver enhancement in gadoxetic acid-enhanced MRI with liver functions: a multicenter-multivendor analysis of hepatocellular carcinoma patients from SORAMIC trial. Eur Radiol..

[50] Schütte K, Schinner R, Fabritius MP, Möller M, Kuhl C, Iezzi R, Öcal O, Pech M, Peynircioglu B, Seidensticker M, Sharma R, Palmer D, Bronowicki JP, Reimer P, Malfertheiner P, Ricke J (2020). Impact of extrahepatic metastases on overall survival in patients with advanced liver dominant hepatocellular carcinoma: a subanalysis of the SORAMIC trial. Liver Cancer..

[51] Uka K, Aikata H, Takaki S, Shirakawa H, Jeong SC, Yamashina K, Hiramatsu A, Kodama H, Takahashi S, Chayama K (2007). Clinical features and prognosis of patients with extrahepatic metastases from hepatocellular carcinoma. World J Gastroenterol..

[52] Venerito M, Pech M, Canbay A, Donghia R, Guerra V, Chatellier G, Pereira H, Gandhi M, Malfertheiner P, Chow PKH, Vilgrain V, Ricke J, Leandro G (2020). NEMESIS: noninferiority, individual-patient metaanalysis of selective internal radiation therapy with 90Y resin microspheres versus sorafenib in advanced hepatocellular carcinoma. J Nucl Med..

[53] Kappadath SC, Mikell J, Balagopal A, Baladandayuthapani V, Kaseb A, Mahvash A (2018). Hepatocellular carcinoma tumor dose response after 90Y-radioembolization with glass microspheres using 90Y-SPECT/CT-based voxel dosimetry. Int J Radiat Oncol Biol Phys..

[54] Lewandowski RJ, Salem R (2021). Radioembolisation with personalised dosimetry: improving outcomes for patients with advanced hepatocellular carcinoma. Lancet Gastroenterol Hepatol..

[55] Kaseb AO, Kappadath SC, Lee SS, Raghav KP, Mohamed YI, Xiao L, Morris JS, Ohaji C, Avritscher R, Odisio BC, Kuban J, Abdelsalam ME, Chasen B, Elsayes KM, Elbanan M, Wolff RA, Yao JC, Mahvash A (2021). A prospective phase II study of safety and efficacy of sorafenib followed by 90Y glass microspheres for patients with advanced or metastatic hepatocellular carcinoma. J Hepatocell Carcinoma..

[56] Bagchi S, Yuan R, Engleman EG (2021). Immune checkpoint inhibitors for the treatment of cancer: clinical impact and mechanisms of response and resistance. Annu Rev Pathol..

[57] Zou W, Chen L (2008). Inhibitory B7-family molecules in the tumour microenvironment. Nat Rev Immunol..

[58] FDA grants accelerated approval to nivolumab and ipilimumab combination for hepatocellular carcinoma. In: FDA. 2020. https://www.fda.gov/drugs/resources-information-approved-drugs/fda-grants-accelerated-approval-nivolumab-and-ipilimumab-combination-hepatocellular-carcinoma. Accessed 31 Jan 2022.

[59] FDA grants accelerated approval to pembrolizumab for hepatocellular carcinoma. In: FDA. 2019. https://www.fda.gov/drugs/fda-grants-accelerated-approval-pembrolizumab-hepatocellular-carcinoma. Accessed 1 Feb 2022.

[60] FDA Approves Atezolizumab Plus Bevacizumab for Liver Cancer. In: National Cancer Institute. 2020. https://www.cancer.gov/news-events/cancer-currents-blog/2020/fda-atezolizumab-bevacizumab-liver-cancer. Accessed 1 Feb 2022.

[61] Phase 3 randomized, open-label, multicenter study of tremelimumab (T) and durvalumab (D) as first-line therapy in patients (pts) with unresectable hepatocellular carcinoma (uHCC): HIMALAYA. In: Journal of Clinical Oncology. https://ascopubs.org/doi/abs/10.1200/JCO.2022.40.4_suppl.379. Accessed 31 Jan 2022.

[62] Finn RS, Qin S, Ikeda M, Galle PR, Ducreux M, Kim T-Y, Kudo M, Breder V, Merle P, Kaseb AO, Li D, Verret W, Derek-Zhen X, Hernandez S, Liu J, Huang C, Mulla S, Wang Y, Lim HY, Zhu AX, Cheng A-L (2020). Atezolizumab plus bevacizumab in unresectable hepatocellular carcinoma. N Engl J Med..

[63] Daniel Grass G, Krishna N, Kim S (2016). The immune mechanisms of abscopal effect in radiation therapy. Current Problems in Cancer..

[64] Rodriguez-Ruiz ME, Rodriguez I, Leaman O, López-Campos F, Montero A, Conde AJ, Aristu JJ, Lara P, Calvo FM, Melero I (2019). Immune mechanisms mediating abscopal effects in radioimmunotherapy. Pharmacol Ther..

[65] Zeng J, Harris TJ, Lim M, Drake CG, Tran PT (2013). Immune modulation and stereotactic radiation: improving local and abscopal responses. Biomed Res Int..

[66] Choi C, Yoo GS, Cho WK, Park HC (2019). Optimizing radiotherapy with immune checkpoint blockade in hepatocellular carcinoma. World J Gastroenterol..

[67] Procureur A, Simonaggio A, Bibault JE, Oudard S, Vano YA (2021). Enhance the immune checkpoint inhibitors efficacy with radiotherapy induced immunogenic cell death: a comprehensive review and latest developments. Cancers (Basel)..

[68] Mortezaee K, Najafi M (2021). Immune system in cancer radiotherapy: resistance mechanisms and therapy perspectives. Crit Rev Oncol Hematol..

[69] Chew V, Lee YH, Pan L, Nasir NJM, Lim CJ, Chua C, Lai L, Hazirah SN, Lim TKH, Goh BKP, Chung A, Lo RHG, Ng D, Filarca RLF, Albani S, Chow PKH (2019). Immune activation underlies a sustained clinical response to Yttrium-90 radioembolisation in hepatocellular carcinoma. Gut..

[70] Estrade F, Lescure C, Muzellec L, Pedrono M, Palard X, Pracht M, Le Sourd S, Rolland Y, Uguen T, Garin E, Edeline J (2020). Lymphocytes and neutrophil-to-lymphocyte ratio variations after selective internal radiation treatment for HCC: a retrospective cohort study. Cardiovasc Intervent Radiol..

[71] Zhan C, Ruohoniemi D, Shanbhogue KP, Wei J, Welling TH, Gu P, Park JS, Dagher NN, Taslakian B, Hickey RM (2020). Safety of combined yttrium-90 radioembolization and immune checkpoint inhibitor immunotherapy for hepatocellular carcinoma. J Vasc Interv Radiol..

[72] Marinelli B, Cedillo M, Pasik SD, Charles D, Murthy S, Patel RS, Fischman A, Ranade M, Bishay V, Nowakowski S, Sung M, Marron T, Lookstein R, Schwartz M, Kim E (2020). Safety and efficacy of locoregional treatment during immunotherapy with nivolumab for hepatocellular carcinoma: a retrospective study of 41 interventions in 29 patients. J Vasc Interv Radiol..

[73] Fenton SE, Kircher SM, Mulcahy MF, Mulcahy MF, Mahalingam D, Salem R, Lewandowski R, Kulik L, Benson AB, Kalyan A (2021). A phase I study of nivolumab (NIVO) in combination with TheraSphere (Yttrium-90) in patients with advanced hepatocellular cancer. JCO.

[74] Tai D, Loke K, Gogna A, Kaya NA, Tan SH, Hennedige T, Ng D, Irani F, Lee J, Lim JQ, Too CW, Ng MCH, Tham CK, Lam J, Koo SL, Chong HS, Goh GB, Huang HL, Venkatanarasimha N, Lo R, Chow PKH, Goh BKP, Chung A, Toh HC, Thng CH, Lim TKH, Yeong J, Zhai W, Chan CY, Choo SP (2021). Radioembolisation with Y90-resin microspheres followed by nivolumab for advanced hepatocellular carcinoma (CA 209–678): a single arm, single centre, phase 2 trial. Lancet Gastroenterol Hepatol..

[75] Maestri M, Pallozzi M, Santopaolo F, Cerrito L, Pompili M, Gasbarrini A, Ponziani FR (2022). Durvalumab: an investigational agent for unresectable hepatocellular carcinoma. Expert Opin Investig Drugs..

[76] Lewandowski RJ, Geschwind JF, Liapi E, Salem R (2011). Transcatheter intraarterial therapies: rationale and overview. Radiology.

[77] Chakravarty R, Dash A, Pillai MR (2012). Availability of yttrium-90 from strontium-90: a nuclear medicine perspective. Cancer Biother Radiopharm.

[78] Radosa CG, Radosa JC, Grosche-Schlee S, Zöphel K, Plodeck V, Kühn JP, Kotzerke J, Hoffmann RT (2019). Holmium-166 radioembolization in hepatocellular carcinoma: feasibility and safety of a new treatment option in clinical practice. Cardiovasc Intervent Radiol..

[79] Smits ML, Nijsen JF, van den Bosch MA, Lam MG, Vente MA, Mali WP, van Het Schip AD, Zonnenberg BA (2012). Holmium-166 radioembolisation in patients with unresectable, chemorefractory liver metastases (HEPAR trial): a phase 1, dose-escalation study. Lancet Oncol..

[80] van Roekel C, Bastiaannet R, Smits MLJ, Bruijnen RC, Braat AJAT, de Jong HWAM, Elias SG, Lam MGEH (2021). Dose-effect relationships of 166Ho radioembolization in colorectal cancer. J Nucl Med..

[81] Tan HY, Wong YH, Kasbollah A, Md Shah MN, Abdullah BJJ, Perkins AC, Yeong CH (2022). Development of neutron-activated samarium-153-loaded polystyrene microspheres as a potential theranostic agent for hepatic radioembolization. Nucl Med Commun..

[82] Eary JF, Collins C, Stabin M, Vernon C, Petersdorf S, Baker M, Hartnett S, Ferency S, Addison SJ, Appelbaum F (1993). Samarium-153-EDTMP biodistribution and dosimetry estimation. J Nucl Med..

[83] Hashikin NA, Yeong CH, Abdullah BJ, Ng KH, Chung LY, Dahalan R, Perkins AC (2015). Neutron activated samarium-153 microparticles for transarterial radioembolization of liver tumour with post-procedure imaging capabilities. PLoS ONE.

[84] Wong YH, Tan HY, Kasbollah A, Abdullah BJJ, Yeong CH (2019). Preparation and in vitro evaluation of neutron-activated, theranostic samarium-153-labeled microspheres for transarterial radioembolization of hepatocellular carcinoma and liver metastasis. Pharmaceutics.

[85] Wong YH, Tan HY, Kasbollah A, Abdullah BJJ, Acharya RU, Yeong CH (2020). Neutron-activated biodegradable samarium-153 acetylacetonate-poly-L-lactic acid microspheres for intraarterial radioembolization of hepatic tumors. World J Exp Med..

[86] Klaassen NJM, Arntz MJ, Gil Arranja A, Roosen J, Nijsen JFW (2019). The various therapeutic applications of the medical isotope holmium-166: a narrative review. EJNMMI Radiopharm Chem..

[87] Lepareur N, Lacœuille F, Bouvry C, Hindré F, Garcion E, Chérel M, Noiret N, Garin E, Knapp FFR (2019). Rhenium-188 labeled radiopharmaceuticals: current clinical applications in oncology and promising perspectives. Front Med (Lausanne)..

[88] Stupp R, Mason WP, van den Bent MJ, Weller M, Fisher B, Taphoorn MJB, Belanger K, Brandes AA, Marosi C, Bogdahn U, Curschmann J, Janzer RC, Ludwin SK, Gorlia T, Allgeier A, Denis Lacombe J, Cairncross G, Eisenhauer E, Mirimanoff RO (2005). Radiotherapy plus concomitant and adjuvant temozolomide for glioblastoma. N Engl J Med..

[89] Bao S, Wu Q, McLendon RE, Hao Y, Shi Q, Hjelmeland AB, Dewhirst MW, Bigner DD, Rich JN (2006). Glioma stem cells promote radioresistance by preferential activation of the DNA damage response. Nature.

[90] Chang CH, Horton J, Schoenfeld D, Salazer O, Perez-Tamayo R, Kramer S, Weinstein A, Nelson JS, Tsukada Y (1983). Comparison of postoperative radiotherapy and combined postoperative radiotherapy and chemotherapy in the multidisciplinary management of malignant gliomas a joint radiation therapy oncology group and eastern cooperative oncology group study. Cancer.

[91] Lawrence YR, Wang M, Dicker AP, Andrews D, Curran WJ, Michalski JM, Souhami L, Yung WK, Mehta M (2011). Early toxicity predicts long-term survival in high-grade glioma. Br J Cancer.

[92] Kennedy AS, Nutting C, Coldwell D, Gaiser J, Drachenberg C (2004). Pathologic response and microdosimetry of (90)Y microspheres in man: review of four explanted whole livers. Int J Radiat Oncol Biol Phys..

[93] Pasciak AS, Manupipatpong S, Hui FK, Gainsburg L, Krimins R, Zink MC, Brayton CF, Morris M, Sage J, Donahue DR, Dreher MR, Kraitchman DL, Weiss CR (2020). Yttrium-90 radioembolization as a possible new treatment for brain cancer: proof of concept and safety analysis in a canine model. EJNMMI Res..

[94] van Es RJ, Nijsen JF, van het Schip AD, Dullens HF, Slootweg PJ, Koole R (2001). Intra-arterial embolization of head-and-neck cancer with radioactive holmium-166 poly(L-lactic acid) microspheres: an experimental study in rabbits. Int J Oral Maxillofac Surg..

[95] Mouli SK, Raiter S, Harris K, Mylarapu A, Burks M, Li W, Gordon AC, Khan A, Matsumoto M, Bailey KL, Pasciak AS, Manupipatpong S, Weiss CR, Casalino D, Miller FH, Gates VL, Hohlastos E, Lewandowski RJ, Kim DH, Dreher MR, Salem R (2021). Yttrium-90 radioembolization to the prostate gland: proof of concept in a canine model and clinical translation. J Vasc Interv Radiol..

[96] Hickey RM, Maslowski JM, Aaltonen ET, Horn JC, Patel A, Sista AK, Gross JS (2020). Yttrium-90 radioembolization in the office-based lab. J Vasc Interv Radiol..

[97] Pöpperl G, Helmberger T, Münzing W, Schmid R, Jacobs TF, Tatsch K (2005). Selective internal radiation therapy with SIR-Spheres in patients with nonresectable liver tumors. Cancer Biother Radiopharm..

[98] Jakobs TF, Hoffmann RT, Poepperl G, Schmitz A, Lutz J, Koch W, Tatsch K, Lubiensky A, Reiser MF, Helmberger T (2007). Mid-term results in otherwise treatment refractory primary or secondary liver confined tumours treated with selective internal radiation therapy (SIRT) using (90)Yttrium resin-microspheres. Eur Radiol..

[99] Mancini R, Carpanese L, Sciuto R, Pizzi G, Golfieri R, Giampalma L, Cappelli A, Galaverni MC, Blotta A, Fiore F, Izzo F, Lastoria S, Mastro A, Di Marzo M, Cagol PP, Gasparini D, Geatti O, Bacchetti S, Pasqual E, Zeuli M, Paoletti G, Garufi C, Cosimelli M (2006). A multicentric phase II clinical trial on intra-arterial hepatic radiotherapy with 90yttrium SIR-spheres in unresectable colorectal liver metastases refractory to iv chemotherapy preliminary results on toxicity and response rates. In Vivo..

[100] Andrews JC, Walker SC, Ackermann RJ, Cotton LA, Ensminger WD, Shapiro B (1994). Hepatic radioembolization with yttrium-90 containing glass microspheres: preliminary results and clinical follow-up. J Nucl Med..

[101] Sato K, Lewandowski RJ, Bui JT, Omary R, Hunter RD, Kulik L, Mulcahy M, Liu D, Chrisman H, Resnick S, Nemcek AA, Vogelzang R, Salem R (2006). Treatment of unresectable primary and metastatic liver cancer with yttrium-90 microspheres (therasphere®): assessment of hepatic arterial embolization. CardioVasc. Intervent. Radiol..

[102] Ogawa F, Mino-Kenudson M, Shimizu M, Ligato S, Lauwers GY (2008). Gastroduodenitis associated with yttrium 90-microsphere selective internal radiation: an iatrogenic complication in need of recognition. Arch Pathol Lab Med..

[103] Crowder CD, Grabowski C, Inampudi S, Sielaff T, Sherman CA, Batts KP (2009). Selective internal radiation therapy-induced extrahepatic injury: an emerging cause of iatrogenic organ damage. Am J Surg Pathol..

[104] Stubbs RS, O'Brien I, Correia MM (2006). Selective internal radiation therapy with 90Y microspheres for colorectal liver metastases: single-centre experience with 100 patients. ANZ J Surg..

[105] Grady ED (1979). Internal radiation therapy of hepatic cancer. Dis Colon Rectum..

[106] Leung TW, Lau WY, Ho SK, Ward SC, Chow JH, Chan MS, Metreweli C, Johnson PJ, Li AK (1995). Radiation pneumonitis after selective internal radiation treatment with intraarterial 90yttrium-microspheres for inoperable hepatic tumors. Int J Radiat Oncol Biol Phys..

[107] Gates VL, Marshall KG, Salzig K, Williams M, Lewandowski RJ, Salem R (2014). Outpatient single-session yttrium-90 glass microsphere radioembolization. J Vasc Interv Radiol..

[108] Aberle S, Kenkel D, Becker AS, Puippe G, Burger I, Schaefer N, Pfammatter T (2020). Outpatient yttrium-90 microsphere radioembolization: assessment of radiation safety and quantification of post-treatment adverse events causing hospitalization. Radiol Med..

[109] Iezzi R, Pompili M, Posa A, Annicchiarico E, Garcovich M, Merlino B, Rodolfino E, Di Noia V, Basso M, Cassano A, Barone C, Gasbarrini A, Manfredi R, Colosimo C (2017). Transradial versus transfemoral access for hepatic chemoembolization: intrapatient prospective single-center study. J Vasc Interv Radiol..

[110] Iezzi R, Posa A, Bilhim T, Guimaraes M (2021). Most common misconceptions about transradial approach in interventional radiology: results from an international survey. Diagn Interv Radiol..

[111] Gabr A, Kallini JR, Gates VL, Hickey R, Kulik L, Desai K, Thornburg B, Marshall K, Salzig K, Williams M, Del Castillo C, Ganger D, Hohlastos E, Baker T, Lewandowski RJ, Salem R (2016). Same-day 90Y radioembolization: implementing a new treatment paradigm. Eur J Nucl Med Mol Imaging..

[112] Li MD, Chu KF, DePietro A, Wu V, Wehrenberg-Klee E, Zurkiya O, Liu RW, Ganguli S (2019). Same-day yttrium-90 radioembolization: feasibility with resin microspheres. J Vasc Interv Radiol..

[113] Calvo A, Tabone M, Carbonatto P, Richetta E, Pellerito R (2019). Radioembolization in a single session using 90-yttrium resin microspheres. J Vasc Interv Radiol..

[114] Gabr A, Ali R, Al Asadi A, Mora R, Mouli S, Riaz A, Salem R, Lewandowski RJ (2019). Technical aspects and practical approach toward same-day Y90 radioembolization in the management of hepatocellular carcinoma. Tech Vasc Interv Radiol..

[115] Elsayed M, Loya M, Galt J, Schuster DM, Bercu ZL, Newsome J, Brandon D, Benenati S, Behbahani K, Duszak R, Sethi I, Kokabi N (2021). Same day yttrium-90 radioembolization with single photon emission computed tomography/computed tomography: An opportunity to improve care during the COVID-19 pandemic and beyond. World J Gastrointest Oncol..

[116] Ezponda A, Rodríguez-Fraile M, Morales M, Vivas I, De La Torre M, Sangro B, Bilbao JI (2020). Hepatic flow redistribution is feasible in patients with hepatic malignancies undergoing same-day work-up angiography and yttrium-90 microsphere radioembolization. Cardiovasc Intervent Radiol..

[117] Keane G, Lam M, de Jong H (2021). Beyond the MAA-Y90 paradigm: the evolution of radioembolization dosimetry approaches and scout particles. Semin Intervent Radiol..

[118] Smits MLJ, Dassen MG, Prince JF, Braat AJAT, Beijst C, Bruijnen RCG, de Jong HWAM, Lam MGEH (2020). The superior predictive value of 166Ho-scout compared with 99mTc-macroaggregated albumin prior to 166Ho-microspheres radioembolization in patients with liver metastases. Eur J Nucl Med Mol Imaging..

[119] Elschot M, Nijsen JF, Lam MG, Smits ML, Prince JF, Viergever MA, van den Bosch MA, Zonnenberg BA, de Jong HW (2014). (^99^m)Tc-MAA overestimates the absorbed dose to the lungs in radioembolization: a quantitative evaluation in patients treated with ^166^Ho-microspheres. Eur J Nucl Med Mol Imaging..

[120] Braat AJAT, Prince JF, van Rooij R, Bruijnen RCG, van den Bosch MAAJ, Lam MGEH (2018). Safety analysis of holmium-166 microsphere scout dose imaging during radioembolisation work-up: A cohort study. Eur Radiol..

[121] Bakker RC, Bastiaannet R, van Nimwegen SA, Barten-van Rijbroek AD, Van Es RJJ, Rosenberg AJWP, de Jong HWAM, Lam MGEH, Nijsen JFW (2020). Feasibility of CT quantification of intratumoural 166Ho-microspheres. Eur Radiol Exp..

[122] van Roekel C, Harlianto NI, Braat AJAT, Prince JF, van den Hoven AF, Bruijnen RCG, Lam MGEH, Smits MLJ (2020). Evaluation of the safety and feasibility of same-day holmium-166 -radioembolization simulation and treatment of hepatic metastases. J Vasc Interv Radiol..

